# Racial and ethnic disparities in smoking prevalence in Israel and the United States: progress to date and prospects for the future

**DOI:** 10.1186/s13584-017-0177-9

**Published:** 2017-10-02

**Authors:** Daniel S. Blumenthal

**Affiliations:** Department of Community Health and Preventive Medicine, Morehouse School of Medicine, 720 Westview Dr. SW, Atlanta, GA 30310 USA

**Keywords:** Tobacco, Smoking, Smoking cessation, Disparities, Lung cancer

## Abstract

An article in this journal in 2016 demonstrated that smoking prevalence among Arab men in Israel is greater than among their Jewish counterparts born in Israel, while the reverse is true among Arab and Jewish Israeli women. This is reflected in lung cancer mortality rates. In the U.S., smoking prevalence in the mid-1960s was 20% higher in African American men than in white men, but has since decreased in both groups, and smoking prevalence in the two groups is now nearly identical. The black-white disparity in lung cancer mortality rates has been reduced by more than half as compared to its zenith in the early 1990s. The strategies employed to achieve these gains will continue to be important going forward, and successful strategies employed in Israel in addressing smoking in the male Arab population will be of increasing interest in the U.S. as its Arab population increases.

## Main text

Smoking prevalence among Arab men in Israel is greater than among their Jewish counterparts born in Israel, while the reverse is true among Arab and Jewish Israeli women. These are among the findings reported by Kalter-Leibovici and colleagues in a paper published in this journal in 2016. [1] The disparities are of signal interest to those of us in the United States who study racial and ethnic health disparities, and each country has much to learn from the other. Other countries can learn from these experiences as well.

Kalter-Leibovici did not address the consequences of the disparities in smoking, but they are reflected in data from the Organization for Economic Cooperation and Development (OECD): Israeli lung cancer mortality is over 60% higher in Arab men than in Jewish men, while it is slightly higher in Jewish women than in Arab women. [2]

In the United States, blacks (or African Americans) are the minority group that suffers the most from health disparities. [3] They represent about 13% of the US population. In the mid-1960s, black men smoked at a rate about 20% higher than white men (60% vs 50%). White and black women smoked at about the same rate: around 35%. In 1964, the first US Surgeon General’s Report on Smoking and Health was published, [4] providing, for the first time, government recognition of the fact that smoking causes lung cancer and damages health in numerous other ways as well.

**Fig. 1:**
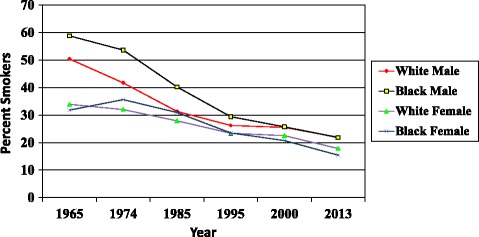
Smoking rates, United States, 1965–2013

That report, and the numerous public health interventions that followed it, are given most of the credit for the subsequent decline in smoking rates in the US; by the mid-1990s, the smoking prevalence in white men had dropped to about 25% and the smoking prevalence among white and black women was a bit lower. Importantly for those concerned about disparities, the smoking rate in black men dropped more rapidly than in white men so that, since 2000, the rate in men of the two races has been almost identical. White and black women continue to smoke at similar rates, with white women currently smoking slightly more than Black women (see Fig. 1).

**Fig. 2:**
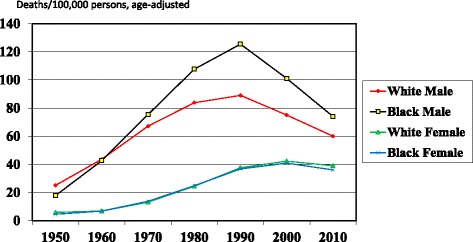
Lung Cancer Mortality Rates, United States, 1950–2010

About 25 years after the publication of the Surgeon General’s report, and after years of steady increase, the lung cancer mortality rates began to drop, and the Black-White disparity began to narrow rapidly (Fig. 2 [5]). In 1990, the gap was about 35 deaths/100,000 population; by 2010 it had narrowed to about 14/100,000. Hence, we should applaud a health promotion success: an intervention (the Surgeon General’s Report) leads to other interventions and a population-wide behavior change (reduced smoking), which in turn leads, 25 years later, to improved health (reduced lung cancer mortality and a reduced disparity).

Israel and the U.S. have employed, or attempted to employ, similar tobacco control measures. These have included, for instance, legislation to limit the number of places where smoking is allowed, health warnings on cigarette packages, and an increase in tobacco taxes, although these and other measures have often met with resistance [2, 6].

The relative success in preventing smoking (and lung cancer mortality) in African Americans has taken place despite efforts by tobacco companies to promote tobacco use in this minority group. [7] Negotiation and legal action have led to the elimination of this targeted promotion. Credit must also be given to smoking prevention and cessation initiatives directed at African Americans, such as the *Pathways to Freedom* program of the Centers for Disease Control and Prevention [8] and efforts of the National African American Tobacco Prevention Network [9] and the American Cancer Society. [10] *Pathways to Freedom*, for instance, features black models in photographs, builds a quit-smoking story around a fictitious black family, includes a section on tobacco marketing in black communities, and offers information on disparities in smoking-related diseases.

An examination of the data in the Kalter-Leibovici paper yields some interesting findings that contrast with the U.S. data. A portion of that paper’s Table 1 is reproduced here and shows that there is only a modest difference between Arab and Jewish men in the ever-smoker rate (past + current smokers), but Jewish men have quit at nearly twice the rate of Arab men. It is this difference in quit rate that accounts for the difference in current-smoking rate.

**Table 1:** Smoking status by population group: men (data from reference [1], Table 1)

	Never *N* = 1704	Past *N* = 844	Current *N* = 1139
Arabs	279 (41.5)	86 (12.8)	307 (45.7)
Jews and others	1424 (47.3)	758 (25.2)	832 (27.6)

## Conclusion

What conclusions can be drawn from this experience? There are several, and they can apply to the U.S., Israel, and other countries as well. First, disparities are not inevitable or uncorrectable, even in low-income minority populations. While social determinants of health are very important as a cause of disparities, interventions directed at a proximate cause of ill health – such as smoking – can be effective. Second, interventions intended to impact minority or disadvantaged populations should be linguistically and culturally tailored, and social service and other organizations representing or reaching out to the targeted group should be enlisted.

Third, the efforts of tobacco companies to promote their products call for constant vigilance on the part of health promotion advocates and government officials. The WHO Framework Convention on Tobacco Control, to which Israel is a signatory, prohibits tobacco advertising, but it must be enforced, and enforcement in Israel is weak. [11] The United States is not a signatory to the Framework Convention, but advertising is very limited under a legal agreement reached in 1998. [12] Fourth, surveillance should be ongoing so that current data are available. It is noteworthy that the Kalter-Leibovici paper that was published in 2016 relied on data collected in 2010. Substantial change in smoking prevalence can take place in a relatively short amount of time; for instance, in the U.S., cigarette smoking overall declined from 20.9% to 15.1% between 2005 and 2015 – a reduction of 27.7%. [13]

Israel’s experience in addressing tobacco use in its Arab population will be of considerable interest in the United States, Canada, and Europe, where the Arab immigrant and refugee population is growing rapidly as a result of war and instability in much of the Middle East. Israel may have an opportunity to provide a model to the West in this regard.

## Abbreviations

OECD: Organization for Economic Cooperation and Development
US: United States
WHO: World Health Organization
